# Assessment of Caffeine Consumption and Maternal Cardiometabolic Pregnancy Complications

**DOI:** 10.1001/jamanetworkopen.2021.33401

**Published:** 2021-11-08

**Authors:** Stefanie N. Hinkle, Jessica L. Gleason, Samrawit F. Yisahak, Sifang Kathy Zhao, Sunni L. Mumford, Rajeshwari Sundaram, Jagteshwar Grewal, Katherine L. Grantz, Cuilin Zhang

**Affiliations:** 1 Department of Biostatistics, Epidemiology and Informatics, Perelman School of Medicine, University of Pennsylvania, Philadelphia; 2 Epidemiology Branch, Division of Intramural Population Health Research, Eunice Kennedy Shriver National Institute of Child Health and Human Development, National Institutes of Health, Bethesda, Maryland; 3 Office of the Director, Division of Intramural Population Health Research, Eunice Kennedy Shriver National Institute of Child Health and Human Development, National Institutes of Health, Bethesda, Maryland; 4 Biostatistics and Bioinformatics Branch, Division of Intramural Population Health Research, Eunice Kennedy Shriver National Institute of Child Health and Human Development, National Institutes of Health, Bethesda, Maryland

## Abstract

**Importance:**

Women are recommended to limit caffeine consumption to less than 200 mg per day based on risks to fetal health. Impacts of caffeine on maternal health remain unclear.

**Objective:**

To determine whether caffeinated-beverage intake and plasma caffeine and paraxanthine are associated with cardiometabolic complications in pregnancy (ie, gestational diabetes [GDM], preeclampsia, and gestational hypertension [GH]).

**Design, Setting, and Participants:**

This cohort study used data from a longitudinal pregnancy cohort study from the National Institute of Child Health and Human Development (NICHD) Fetal Growth Studies-Singletons (2009-2013). This post hoc secondary analysis of 2802 pregnant women without major chronic conditions enrolled at 12 US clinical sites was completed in 2021. The final sample for caffeinated beverage analyses included 2583 women. After excluding women who did not consent to have their biospecimens stored for future research (n = 54), plasma caffeine analyses included 2529 women. Analyses of caffeine consumption and fasting cardiometabolic profiles included 319 women.

**Exposures:**

Daily total caffeine intake was estimated at 10 to 13 gestational weeks and 16 to 22 gestational weeks based on self-reported past week intake of caffeinated coffee, tea, soda, and energy drinks. Plasma caffeine and paraxanthine were measured in specimens collected at 10 to 13 weeks.

**Main Outcomes and Measures:**

Clinical diagnoses of GDM, preeclampsia, GH, glucose concentrations from GDM screening, and blood pressure were extracted from medical records.

**Results:**

Participants had a mean (SD) age of 28.1 (5.5) years and 422 participants (16.3%) were Asian/Pacific Islander women, 741 (28.9%) were Hispanic women, 717 (27.8%) were non-Hispanic Black women, and 703 (27.2%) were non-Hispanic White women. At 10 to 13 weeks, 1073 women (41.5%) reported consuming no caffeinated beverages, 1317 (51.0%) reported consuming 1 mg/d to 100 mg/d, 173 (6.7%) reported consuming 101 mg/d to 200 mg/d, and 20 (0.8%) reported consuming more than 200 mg/d. At 16 to 22 weeks, 599 women (23.6%) reported consuming no caffeinated beverages, 1734 (68.3%) reported consuming 1 mg/d to 100 mg/d, 186 (7.3%) reported consuming 101 mg/d to 200 mg/d, and 20 (0.8%) reported consuming more than 200 mg/d caffeinated beverages. Intake at 16 to 22 weeks was associated with lower GDM risk and lower glucose concentrations (1 mg/d to 100 mg/d vs none: relative risk, 0.53 [95% CI, 0.35 to 0.80]; β, –2.7 mg/dL [95% CI, –5.4 mg/dL to 0 mg/dL]) and lower C-reactive protein and C-peptide concentrations and favorable lipid profiles. Total plasma caffeine and paraxanthine at 10 to 13 weeks was inversely associated with glucose (quartile 4 vs quartile 1: β = –3.8 mg/dL [95% CI, –7.0 mg/dL to –0.5 mg/dL]; trend of *P* = .01). No associations were observed with preeclampsia or GH.

**Conclusions and Relevance:**

In this cohort study, second trimester caffeinated beverage intake within current recommendations was associated with lower GDM risk, but not preeclampsia or GH. These findings may be reassuring for women with moderate caffeine intake.

## Key Points

**Question:**

Is caffeine intake associated with major cardiometabolic complications during pregnancy (ie, gestational diabetes [GDM], preeclampsia, gestational hypertension)?

**Findings:**

In this cohort study of 2802 pregnant women, low and moderate caffeinated beverage intake early in second trimester within current guidelines of less than 200 mg per day were associated with a lower risk for GDM, lower glucose levels at GDM screening, and more favorable cardiometabolic profile compared with no consumption. Caffeine was not associated with gestational hypertension or preeclampsia.

**Meaning:**

The findings of this study may be reassuring for pregnant women with moderate caffeine intake and should be considered in the context of published data on associations with offspring health.

## Teaser

This cohort study examines whether caffeinated-beverage intake and plasma caffeine and paraxanthine are associated with cardiometabolic complications in pregnancy.

## Introduction

Over 80% of US women of reproductive age consume caffeine daily.^1^ While most women decrease consumption after becoming pregnant, many continue to consume caffeine throughout pregnancy.^2^ The American College of Obstetricians and Gynecologists (ACOG) recommends that pregnant women limit their caffeine consumption to less than 200 mg/d out of an abundance of caution because of potential associations with pregnancy loss and fetal growth restriction at higher intakes.^3^ There remains limited data on associations with maternal cardiometabolic outcomes in pregnancy.

Outside of pregnancy, caffeine (or coffee) is associated with both acute and chronic cardiometabolic changes.^4^ Among nonhabitual consumers, caffeine acutely raises blood pressure.^5^ Nevertheless, among women who were habitual consumers, increasing coffee consumption was associated with lower hypertension risk in prospective cohort studies.^6^ Experimental studies demonstrate that acute caffeine intake reduces insulin sensitivity,^7^ yet there are limited long-term effects.^8^ Observational studies have observed inverse associations between habitual coffee intake and type 2 diabetes.^9^ Given that pregnancy is a period with increasing insulin resistance^10^ and reduced caffeine metabolism,^11^ there is a need to study caffeine in pregnancy with maternal cardiometabolic health, specifically.

Previous studies on caffeine (or coffee) and gestational diabetes (GDM),^12,13^ gestational hypertension,^14^ and preeclampsia^15^ were null except for an inverse association between moderate intakes and preeclampsia.^14^ These studies were limited by lack of multiple assessments across pregnancy,^12,13,15^ objective measures,^12,13,14^ and limited lifestyle measures to reduce confounding.^13,15^

This study aimed to examine prospective associations between caffeine exposure in early- and mid-pregnancy and maternal cardiometabolic health, including GDM, preeclampsia, and gestational hypertension as well as cardiometabolic biomarkers and blood pressure.

## Methods

Institutional review board approval was obtained at the clinical sites, data coordinating center, and the Eunice Kennedy Shriver National Institute of Child Health and Human Development (NICHD). All participants provided written informed consent. This study followed the Strengthening the Reporting of Observational Studies in Epidemiology (STROBE) reporting guidelines.

### Study Population

This was a secondary analysis of the NICHD Fetal Growth Studies-Singleton Cohort (n = 2802).^16^ The primary aim was to establish race-specific and ethnicity-specific US standards for fetal growth.^17^ Women of underrepresented minority groups were oversampled and enrolled based on their self-identified classification into one of the following categories: Asian/Pacific Islander, Hispanic, non-Hispanic Black, and non-Hispanic White. Women were enrolled between gestational weeks 8 to 13 at 12 US clinical centers (2009-2013). Eligibility among women with a prepregnancy body mass index (BMI; calculated as weight in kilograms divided by height in meters squared) of 19.0 to 29.9 was limited to women who did not smoke, who were not currently drinking 1 or more alcoholic drinks per day or using illicit drugs, who conceived without fertility drugs or in vitro fertilization, who did not have prior pregnancy complications, and who did not have major chronic diseases. Eligibility among women with a prepregnancy BMI of 30.0 to 45.0 was limited to women without major chronic diseases. Complete details are published elsewhere.^16^ Statistical analyses for the current study were completed in 2021.

Questionnaire data, research examinations, and biospecimens were collected at enrollment, throughout pregnancy, and at delivery. Research visits were targeted at 16 to 22 (visit 1), 24 to 29 (visit 2), 30 to 33 (visit 3), and 34 to 37 weeks (visit 4). Medical record abstraction of routine prenatal examinations and delivery discharge diagnoses was completed after delivery. Biospecimens were collected at enrollment, visits 1, 2, and 4 following a standardized protocol, immediately processed into plasma, and stored at –80°C.

The study sample is described in eFigure 1 in the Supplement. Eighteen women were determined to be ineligible after enrollment and excluded from the analysis. Women who exited the study or were without medical record abstraction were excluded (201; 7%); no differences in caffeinated beverage intake (*P* = .18) or plasma caffeine (*P* = .54) were observed between women with and without medical record abstraction. The final sample for caffeinated beverage analyses included 2583 women. After excluding women who did not consent to have their biospecimens stored for future research (n = 54), plasma caffeine analyses included 2529 women. Analyses of caffeinated beverage intake and fasting cardiometabolic profile were based on a subsample of 319 women by leveraging biomarker data from 16 to 22 weeks previously measured as part of a GDM case-control study (107 GDM cases; 214 non-GDM controls matched 2:1 on age, race and/or ethnicity, gestational week of blood collection; 2 controls excluded because of missing medical record abstraction).^18^

### Exposure Ascertainment

At enrollment and each visit thereafter, women reported past week intake of cups (8 oz) of caffeinated coffee, cups (8 oz) of caffeinated tea, cans (12 oz) or bottles (16 oz) of soda with caffeine (eg, Coke, Pepsi, Dr Pepper, Mountain Dew), and cans (12 oz) or bottles (16 oz) of energy drinks with caffeine (eg, Red Bull, Amp). Participants who reported drinking less than one serving (eg, cup, can, or bottle) per day were classified as drinking half a serving. Total daily caffeine was estimated by multiplying servings of each beverage by estimated caffeine content (coffee, 96 mg per 8oz; tea, 48 mg per 8oz; soda, 40 mg per 12 oz; energy drink, 108 mg per 12 oz) and summing across beverages.^19^

Concentrations of caffeine and paraxanthine were measured in plasma collected at 10 to 13 weeks’ gestation by a hybrid solid-phase extraction, and quantification of caffeine was performed on an ABSCIEX 5500 (Applied Biosystems).^20^ Caffeine and paraxanthine detection limits were 0.55 ng/mL and 0.72 ng/mL and limits of quantification were 1.85 ng/mL and 2.39 ng/mL, respectively. Machine observed values were used if values were below limits of detection or quantification to minimize bias.^21,22^ Total methylxanthine concentrations were estimated as the sum of caffeine and paraxanthine.

### Outcome Ascertainment

Primary outcomes were clinical diagnoses of GDM, gestational hypertension, preeclampsia, glucose (from glucose challenge test [GCT]), and blood pressure extracted from medical records.^23^ All women received routine prenatal care. The majority of women (90%) were screened for GDM using a 2-step process with 50-g GCT (median 27 weeks) and 100-g oral glucose tolerance test (OGTT), as necessary. Women were classified with GDM based on OGTT results using Carpenter-Coustan criteria (ie, at least 2 elevated plasma glucose values: fasting, 95 mg/dL; 1-hour, 180 mg/dL; 2-hour, 155 mg/dL; 3-hour, 140 mg/dL; to convert glucose to millimoles per liter, multiply by 0.0555), and/or receipt of GDM medications.^24,25^ Impaired glucose tolerance (IGT) was defined as a 2-hour OGTT plasma glucose concentration between 140 mg/dL to 199 mg/dL, but not meeting criteria for GDM.^25^

Secondary outcomes included the following plasma fasting cardiometabolic biomarkers measured at 16 to 22 weeks: C-reactive protein, glucose, insulin, C-peptide, high-density lipoproteins (HDL), total cholesterol, triglycerides, low-density lipoproteins (LDL), and HbA1c (eTable 1 in the Supplement). Homeostatic model assessment for insulin resistance (HOMA-IR) was calculated as: Insulin[mg/dL]×Glucose[mg/dL])/405.^26,27^

### Covariates

Accurate gestational dating by last menstrual period was confirmed by ultrasonography at enrollment. At enrollment, women self-reported their medical history and sociodemographics. Prepregnancy BMI was calculated from the measured enrollment height and self-reported prepregnancy weight. A family history of diabetes was classified if a woman’s parents or siblings had diabetes. Moderate and vigorous physical activity (metabolic hr/wk) was estimated at enrollment (ie, prior year activity) and at each visit thereafter (ie, activity since prior visit).^28^ Past month perceived stress was estimated at enrollment and each visit using the Perceived Stress Scale.^29^ Habitual dietary intake over past 3 months (ie, periconception and first trimester) was assessed at enrollment using a food frequency questionnaire.^30^ Dietary quality was assessed using the Healthy Eating Index (HEI)-2010 total score.^31^ Plasma cotinine was measured using ultraperformance liquid chromatography coupled with electrospray triple-quadrupole tandem mass spectrometry.

### Statistical Analysis

Caffeinated beverage intake was assessed as categories (ie, 0, 1-100, 101-200, more than 200 mg/d) and per 50 mg/d. Plasma caffeine, paraxanthine, and their sum were assessed as quartiles and per 100 μg/L. Participant characteristics were compared by caffeinated beverage intake at enrollment using analysis of variance or χ^2^ tests as applicable.

Prospective associations between caffeine and GDM risk were estimated using Poisson regression with robust error variance^32^; associations with continuous glucose were estimated using linear regression. For gestational hypertension and preeclampsia, multilevel outcomes necessitated that associations were estimated using multinomial logistic regression. Because gestational hypertension and preeclampsia were relatively rare (ie, less than 4%), odds ratios should approximate risk ratios.^33^ Only caffeinated beverage intake reported at 10 to 13 weeks and 16 to 22 weeks were used to ensure that analyses were prospective and exposures were measured before a diagnosis of GDM, gestational hypertension, or preeclampsia. Caffeine and paraxanthine were summed as an indicator of total consumption and examined individually to assess for differences in associations by the metabolites themselves.^34^

A linear trend test across quartiles of plasma caffeine and paraxanthine was performed using the median value within each quartile estimated as a continuous exposure. Covariates for primary models were selected a priori to include age, prepregnancy BMI, race and/or ethnicity, parity, marital status, and education. Sensitivity analyses additionally adjusted for any of the following covariates if they were significantly associated with exposure or outcome in bivariate analyses: employment, family history of diabetes, severe nausea or hyperemesis, prepregnancy alcohol intake, stress, sleep, moderate and vigorous physical activity, cotinine, total energy intake, and HEI-2010 total score. The majority of women in the study did not smoke; sensitivity analyses excluded women who reported smoking 6 months before enrollment (n = 17). To increase power, sensitivity analyses combined IGT and GDM. Missing exposure, covariate, and GCT glucose data were multiply imputed with 20 replicates.^35^

Blood pressure trajectories across gestation were estimated by caffeine exposures at 10 to 13 weeks using linear mixed-effects models with cubic splines. Three-knot points (ie, 25th, 50th, 75th percentiles) were chosen at gestational weeks 17, 28, and 35 that evenly split the distributions. *P* values for exposures were estimated using a likelihood ratio test. Blood pressure across gestation was plotted for each exposure at the mean sample covariate distribution; covariates included age, prepregnancy BMI, race and/or ethnicity, parity, marital status, and education.

Prospective associations between caffeine intake and fasting cardiometabolic biomarkers were assessed at 16 to 22 weeks. All outcomes were log-transformed. Associations were estimated using linear regression with results presented as percent change in outcome. Because biomarker data was leveraged from a prior GDM case-control study, analyses were weighted using inverse probability weights to represent the full cohort, and standard errors were estimated using robust error variance. Models included all matching factors for the original case-control study (ie, age, race and/or ethnicity, gestational week at blood collection).^36,37^ Additional covariates included prepregnancy BMI, parity, marital status, and education. Missing exposure, covariate, and biomarker data (because of sample volume) were multiply imputed with 20 replicates.^35^ Analyses were 2-sided with a level of significance of *P* < .05. All analyses were performed using SAS version 9.4 (SAS Institute) from October 2020 to August 2021.

## Results

Participants had a mean (SD) age of 28.1 (5.5) years and 422 participants (16.3%) were Asian/Pacific Islander women, 741 (28.9%) were Hispanic women, 717 (27.8%) were non-Hispanic Black women, and 703 (27.2%) were non-Hispanic White women. Of participants between 10 to 13 gestational weeks at enrollment, 1510 women (58.5%) reported consuming any caffeinated beverages in the past week (soda: 833 [58.5%]; coffee: 613 [40.6%]; tea: 485 [32.1%]; energy drinks: 8 [0.5%]). Race and/or ethnicity, parity, education, marital status, alcohol, stress, cotinine, and HEI-2010 total score differed across caffeine intake (Table 1). At 16 to 22 gestational weeks, 1940 women (76.4%) reported consuming any caffeinated beverages in the past week, 1789 (71.9%) at 24 to 29 weeks, 1710 (69.8%) at 30 to 33 weeks, and 1593 (69.6%) at 34 to 37 weeks. Median (IQR) concentration of plasma caffeine was 169.1 ng/mL (29.5-651.1 ng/mL), and the median (IQR) concentration of paraxanthine was and 74.4 ng/mL (15.2-235.8 ng/mL). Participant characteristics by total plasma caffeine and paraxanthine are shown in eTable 2 in the Supplement. The Spearman correlation between past-week self-reported total caffeinated beverage intake and plasma caffeine and paraxanthine was 0.47 and 0.48, respectively.

**Table 1.** Characteristics of Study Participants According to Caffeinated Beverage Intake Reported at 10 to 13 Weeks, NICHD Fetal Growth Studies–Singleton Cohort (N = 2583)

Characteristics^a^	Participants, No. (%)					
	Past week caffeinated beverage intake, mg/d					
	Overall (n = 2583)	0 (n = 1073)	1-100 (n = 1317)	101-200 (n = 173)	>200 (n = 20)	*P* value^b^
Age, mean (SD), y	28.1 (5.5)	28 (5.4)	28.3 (5.5)	28 (5.7)	25.6 (4.8)	.08
Race/ethnicity						
Asian/Pacific Islander	422 (16.3)	184 (17.2)	224 (17.4)	13 (7.5)	1 (5.0)	.002
Hispanic	741 (28.9)	299 (27.9)	375 (26.4)	62 (35.8)	5 (25.0)	
Non-Hispanic Black	717 (27.8)	323 (30.1)	339 (27.6)	45 (26.0)	10 (50.0)	
Non-Hispanic White	703 (27.2)	267 (24.9)	379 (28.7)	53 (30.6)	4 (20.0)	
Prepregnancy BMI, mean (SD)	25.4 (5.1)	25.3 (5.0)	25.4 (5.2)	26.1 (5.6)	25.4 (4.8)	.22
Nulliparous, No.	1208 (46.8)	548 (51.1)	588 (44.7)	65 (37.6)	7 (35.0)	<.001
Education						
High school or less	770 (29.8)	305 (28.4)	390 (29.6)	63 (36.4)	12 (60.0)	.002
Some college or associate degree	777 (30.1)	346 (32.3)	371 (28.2)	55 (31.8)	5 (25.0)	
4-y college degree or higher	1036 (40.1)	422 (39.3)	556 (42.2)	55 (31.8)	3 (15.0)	
Married	1920 (74.4)	789 (73.6)	1005 (76.4)	121 (69.9)	5 (25.0)	<.001
Full-time job or student	1825 (70.7)	769 (71.7)	916 (69.6)	125 (72.3)	15 (75.0)	.62
Family history of diabetes	537 (21.5)	231 (22.2)	267 (21.0)	37 (21.6)	2 (10.0)	.56
Severe vomiting or hyperemesis, first trimester	126 (4.9)	56 (5.2)	61 (4.6)	6 (3.5)	3 (15.0)	.13
Alcohol intake prior to pregnancy, median (IQR), No. of drinks per day	0 (0-4.3)	0 (0-0.1)	0.1 (0-4.3)	0 (0-4.3)	0.1 (0-4.3)	<.001
Moderate and vigorous physical activity, past year, median (IQR), MET hr/wk	96.3 (51.4 169.7)	96.7 (49.9-167.9)	94.4 (52.9-169.2)	105.2 (52-181.8)	116.9 (75.6-207.8)	.57
Perceived stress scale score, mean (SD)	11.5 (6.3)	11.2 (6.1)	11.6 (6.3)	12.9 (6.4)	14.0 (6.7)	.002
Sleep, mean (SD), h	8.2 (1.5)	8.1 (1.5)	8.2 (1.5)	8.0 (1.5)	8.5 (1.7)	.21
Cotinine, median (IQR), ng/mL	0 (0-0.1)	0 (0-0.1)	0 (0-0.1)	0 (0-0.2)	0 (0-0.9)	.011
Total energy, past 3 months, median (IQR), kcal/d	1897 (1434-2614)	1926 (1410-2614)	1867 (1451-2595)	2046 (1523-2981)	1709 (1186-1849)	.39
Healthy Eating Index–2010 total score, mean (SD)	65.0 (10.3)	66.2 (10.2)	64.5 (10.2)	61.6 (9.9)	56.3 (9.3)	<.001
Caffeinated soda, median (IQR), mg/d	0 (0-20.0)	0 (0)	20.0 (0-20.0)	20.0 (20.0-80.0)	30.0 (0-120.0)	<.001
Caffeinated coffee, median (IQR), mg/d	0 (0)	0 (0)	0 (0-48.0)	96.0 (0-96.0)	96.0 (0-144.0)	<.001
Caffeinated tea, mg/d	0 (0)	0 (0)	0 (0-24.0)	0 (0-24.0)	0 (0-144.0)	<.001
Caffeinated energy drink, median (IQR), mg/d	0 (0)	0 (0)	0 (0)	0 (0)	0 (0)	<.001

Abbreviations: BMI, body mass index calculated as weight in kilograms divided by height in meters squared; MET, metabolic equivalent of task; NICHD, National Institute of Child Health and Human Development.

SI conversion factor: To convert cotinine to nmol/L, multiply by 5.675.

^a^ Missing covariates: prepregnancy BMI, n = 19; employment, n = 1; marital status, n = 2; family history of diabetes, n = 82; alcohol intake, n = 1; physical activity, n = 6; stress, n = 23; sleep, n = 3; cotinine, n = 152; total energy, n = 1026; Healthy Eating Index–2010 total score, n = 1026.

^b^ Differences in continuous covariates was assessed using a 1-way analysis of variance for normally distributed continuous variables, the Kruskal-Wallis test for nonparametric continuous variables, and χ^2^ test for categorical variables.

At 10 to 13 weeks, 1073 women (41.5%) reported consuming no caffeinated beverages, 1317 (51.0%) reported consuming 1 mg/d to 100 mg/d, 173 (6.7%) reported consuming 101 mg/d to 200 mg/d, and 20 (0.8%) reported consuming more than 200 mg/d. Caffeinated beverage intake at 10 to 13 gestational weeks was not related to GDM risk (Table 2). At 16 to 22 weeks, 599 women (23.6%) reported consuming no caffeinated beverages, 1734 (68.3%) reported consuming 1 mg/d to 100 mg/d, 186 (7.3%) reported consuming 101 mg/d to 200 mg/d, and 20 (0.8%) reported consuming more than 200 mg/d caffeinated beverages. Caffeinated beverage intake of 1 mg/d to 100 mg/d at 16 to 22 weeks was associated with a 47% reduction in GDM risk compared with no intake (relative risk [RR], 0.53 [95% CI, 0.35 to 0.80]); estimate for 101 mg/d to 200 mg/d were similar in magnitude but imprecise (RR, 0.54 [95%CI, 0.24 to 1.18]). Caffeinated beverage intakes of 1 mg/d to 100 mg/d and 101 mg/d to 200 mg/d were associated with lower risk of combined outcome of GDM or IGT (eTable 3 in the Supplement). Intake of 1 mg/d to 100 mg/d at 16 to 22 weeks was associated with lower glucose concentrations by –2.7 mg/dL (95% CI, –5.4 mg/dL to 0 mg/dL) (Table 2). Associations were consistent in magnitude across sensitivity analyses (eTable 4 in the Supplement).

**Table 2.** Self-reported Caffeine Intake and Associations With Gestational Diabetes Risk and Continuous Glucose From the Glucose Challenge Test (N = 2583)

Characteristics	Past week caffeinated beverage intake, mg/d									
	Relative risk for gestational diabetes, (95% CI)^a^					Glucose challenge test results, mg/dL (95% CI)^b^				
	0	1-100	101-200	>200	Per 50	0	1-100	101-200	>200	Per 50
10-13 wk										
Unadjusted	1.00 [Reference]	0.78 (0.53 to 1.16)	1.03 (0.50 to 2.15)	1.16 (0.17 to 7.93)	1.01 (0.84 to 1.21)	0 [Reference]	–1.3 (–3.7 to 1.1)	0.7 (–4.1 to 5.5)	–1.2 (–14.4 to 11.9	0.0 (–1.1 to 1.2)
Adjusted^c^	1.00 [Reference]	0.71 (0.48 to 1.05)	0.97 (0.47 to 1.99)	1.87 (0.23 to 14.89)	0.98 (0.78 to 1.23)	0 [Reference]	–1.8 (–4.1 to 0.5)	0.2 (–4.4 to 4.8)	1.1 (–11.5 to 13.7)	–0.2 (–1.3 to 1.0)
16-22 wk										
Unadjusted	1.00 [Reference]	0.57 (0.38 to 0.87)	0.67 (0.30 to 1.49)	NA^d^	0.82 (0.63 to 1.07)	0 [Reference]	–2.2 (–5.0 to 0.6)	–3.0 (–7.9 to 1.9)	–3.1 (–15.9 to 9.6)	–0.6 (–1.8 to 0.7)
Adjusted^c^	1.00 [Reference]	0.53 (0.35 to 0.80)	0.54 (0.24 to 1.18)	NA^d^	0.75 (0.57 to 0.98)	0 [Reference]	–2.7 (–5.4 to 0.0)	–4.4 (–9.1 to 0.3)	–1.4 (–13.6 to 10.9)	–1.1 (–2.3 to 0.1)

Abbreviations: GDM, gestational diabetes; NA, not applicable.

^a^ Relative risks estimated using a log Poisson model with robust variance with covariates multiply imputed (M = 20).

^b^ Continuous glucose challenge test results estimated using linear regression model with covariates and outcome multiply imputed (M = 20). To convert glucose to mmol/L, multiply by 0.0555.

^c^ Models adjusted for age, prepregnancy BMI, race/ethnicity, education, marital status, and nulliparity.

^d^ Women with caffeine intake more than 200 mg/d were excluded because of lack of model convergence due to a lack of GDM cases within this category.

Plasma caffeine and paraxanthine at 10 to 13 weeks were not associated with GDM (Table 3) nor GDM or IGT risk (eTable 5 in the Supplement). Glucose concentrations were 3.8 mg/dL (–7.0 mg/dL to –0.5 mg/dL) lower among women with total caffeine and paraxanthine in the fourth vs first quartile (trend for *P* = .01) (Table 3). Associations were consistent across sensitivity analyses (eTable 6 in the Supplement).

**Table 3.** Plasma Caffeine Metabolites at 10-13 Weeks’ Gestation and Associations With Gestational Diabetes Risk and Continuous Glucose Measured From the Glucose Challenge Test (N = 2529)

10-13 wk	Relative risk for gestational diabetes (95% CI)^a^^,^^b^						Glucose challenge test results, mg/dL (95% CI)^a^^,^^c^					
	Quartile 1	Quartile 2	Quartile 3	Quartile 4	*P* for trend	Per 100 ug/dL	Quartile 1	Quartile 2	Quartile 3	Quartile 4	*P* for trend	Per 100 ug/dL
Caffeine^d^												
Unadjusted	1.00 [Reference]	0.83 (0.45 to 1.54)	0.99 (0.56 to 1.74)	0.92 (0.52 to 1.64)	.95	0.99 (0.97 to 1.01)	0.0 [Reference]	–1.6 (–5.1 to 1.9)	–0.2 (–3.6 to 3.3)	–2.5 (–5.9 to 0.9)	.16	–0.1 (–0.2 to 0.0)
Adjusted	1.00 [Reference]	0.88 (0.48 to 1.62)	0.95 (0.55 to 1.65)	0.83 (0.45 to 1.51)	.61	0.98 (0.96 to 1.01)	0.0 [Reference]	–1.4 (–4.7 to 2.0)	–0.4 (–3.7 to 3.0)	–3.7 (–7.1 to –0.4)	.02	–0.1 (–0.2 to 0.0)
Paraxanthine^e^												
Unadjusted	1.00 [Reference]	0.91 (0.50 to 1.64)	1.15 (0.65 to 2.01)	1.05 (0.58 to 1.89)	.74	1.00 (0.96 to 1.05)	0.0 [Reference]	–1.5 (–4.8 to 1.8)	1.0 (–2.4 to 4.5)	–2.9 (–6.4 to 0.5)	.07	–0.3 (–0.7 to 0.0)
Adjusted	1.00 [Reference]	0.93 (0.53 to 1.66)	1.11 (0.64 to 1.93)	0.89 (0.49 to 1.64)	.77	0.98 (0.93 to 1.03)	0.0 [Reference]	–1.1 (–4.3 to 2.1)	1.0 (–2.4 to 4.3)	–4.3 (–7.6 to –1.0)	.004	–0.6 (–0.9 to –0.2)
Total caffeine plus paraxanthine^f^												
Unadjusted	1.00 [Reference]	0.93 (0.53 to 1.65)	1.02 (0.57 to 1.82)	1.01 (0.57 to 1.76)	.90	0.99 (0.98 to 1.01)	0.0 [Reference]	–0.9 (–4.3 to 2.4)	–0.5 (–4.0 to 3.0)	–2.4 (–5.7 to 1.0)	.15	–0.1 (–0.2 to 0.0)
Adjusted	1.00 [Reference]	0.94 (0.53 to 1.66)	1.01 (0.57 to 1.78)	0.87 (0.48 to 1.56)	.66	0.99 (0.97 to 1.01)	0.0 [Reference]	–0.8 (–4.0 to 2.5)	–0.4 (–3.8 to 3.0)	–3.8 (–7.0 to –0.5)	.01	–0.1 (–0.2 to 0.0)

^a^ Models adjusted for age, prepregnancy BMI, race/ethnicity, education, marital status, and nulliparity.

^b^ Relative risks estimated using a log Poisson model with robust variance with missing exposure and covariates multiply imputed (M = 20).

^c^ Continuous glucose challenge test results estimated using linear regression model with exposure, covariates, and outcome multiply imputed (M = 20). To convert glucose to mmol/L, multiply by 0.0555.

^d^ The range for plasma caffeine in each quartile was: −2.4 μg/dL to 29.4 μg/dL; 29.5 μg/dL to 160.7 μg/dL; 160.9 μg/dL to 645.8 μg/dL; 646.8 μg/dL to 12489.7 μg/dL.

^e^ The range for plasma paraxanthine in each quartile was: −2.6 μg/dL to 15.6 μg/dL; 15.7 μg/dL to 73.6 μg/dL; 73.6 μg/dL to 234.4 μg/dL; 234.4 μg/dL to 6145.2 μg/dL.

^f^ The range for total plasma caffeine and paraxanthine in each quartile was: −5.0 μg/dL to 48.7 μg/dL; 48.8 μg/dL to 243.0 μg/dL; 243.3 μg/dL to 903.6 μg/dL; 905.7 μg/dL to 13 269.8 μg/dL.

Neither caffeinated beverage intake (Table 4) nor plasma caffeine or paraxanthine were associated with gestational hypertension (83 [3.1%]) or preeclampsia (99 [3.8%]) (eTable 7 to eTable8 in the Supplement). No significant differences in blood pressure across gestation were observed for beverage intake or plasma caffeine and paraxanthine (eFigure 2 in the Supplement). Caffeinated beverage intake at 16 to 22 weeks was associated with some improvements in fasting cardiometabolic profile (Table 5).

**Table 4.** Self-reported Caffeinated Beverage Intake and Associations With Gestational Hypertension and Preeclampsia (N = 2583)

Characteristics	Past week caffeinated beverage intake, mg/d									
	Odds ratio for gestational hypertension (95% CI)^a^					Odds ratio for preeclampsia (95% CI)^a^				
	0	1-100	101-200	>200	Per 50	0	1-100	101-200	>200	Per 50
10-13 wk										
Unadjusted	1.00 [Reference]	0.91 (0.57-1.45)	1.06 (0.44-2.56)	1.60 (0.21-12.38)	0.99 (0.79-1.25)	1.00 [Reference]	1.08 (0.70-1.67)	0.84 (0.32-2.16)	NA^b^	0.94 (0.75-1.19)
Adjusted^c^	1.00 [Reference]	0.96 (0.59-1.55)	0.97 (0.39-2.42)	1.41 (0.17-11.70)	1.00 (0.79-1.28)	1.00 [Reference]	1.22 (0.78-1.91)	0.82 (0.31-2.15)	NA^b^	0.97 (0.77-1.23)
16-22 wk										
Unadjusted	1.00 [Reference]	1.01 (0.59-1.72)	1.05 (0.41-2.67)	NA^b^	0.88 (0.67-1.15)	1.00 [Reference]	0.86 (0.52-1.40)	1.11 (0.49-2.53)	2.56 (0.56-11.68)	1.09 (0.89-1.34)
Adjusted^c^	1.00 [Reference]	1.17 (0.67-2.03)	1.04 (0.39-2.75)	NA^b^	0.90 (0.69-1.18)	1.00 [Reference]	0.99 (0.59-1.64)	1.23 (0.52-2.89)	2.74 (0.56-13.30)	1.15 (0.94-1.42)

Abbreviation: NA, not applicable.

^a^ Odds ratios estimated using multinomial logistic regression models with exposures and covariates multiply imputed (M = 20).

^b^ Women with caffeine intake more than 200 mg/d excluded due to lack of model convergence due to limited cases of preeclampsia or gestational hypertension within this category.

^c^ Models adjusted for a priori covariates of age, prepregnancy BMI, race/ethnicity, education, marital status, and nulliparity.

**Table 5.** Self-reported Past Week Caffeinated Beverage Intake at 16-22 Weeks and Fasting Cardiometabolic Biomarkers at 16-22 Weeks (N = 319)^a^

Cardiometabolic biomarkers at 16-22 weeks^b^	Self-reported caffeinated beverage intake at 16-22, median (IQR)			
	0 mg/d	1-100 mg/d	101-200 mg/d	>200 mg/d
C-Peptide, median (IQR), nmol/L	0.6 (0.4 to 0.7)	0.6 (0.4 to 0.8)	0.5 (0.4 to 0.6)	0.5 (0.4 to 0.6)
% Difference (95% CI)	0.0 [Reference]	–1.9 (–16.2 to 14.9)	–17.7 (–30.3 to –2.9)	–14.6 (–44.9 to 32.5)
HbA_1c_, median (IQR), %	5.0 (4.9 to 5.3)	5.0 (4.7 to 5.2)	5.1 (4.7 to 5.3)	4.8 (4.8 to 5.1)
% Difference (95% CI)	0.0 [Reference]	–1.1 (–4.0 to 1.8)	–0.6 (–4.4 to 3.4)	0.8 (–2.7 to 4.4)
Glucose, median (IQR), md/dL	84.0 (80.0 to 90.0)	84.0 (78.0 to 92.0)	84.0 (80.0 to 89.0)	80.0 (75.0 to 83.0)
% Difference (95% CI)	0.0 [Reference]	–0.1 (–2.8 to 2.7)	–0.7 (–3.4 to 2.1)	–4.5 (–11.9 to 3.6)
HOMA-IR, median (IQR)	1.8 (1.0 to 2.9)	2.0 (1.1 to 3.4)	1.3 (1.0 to 2.0)	1.1 (1.1 to 1.1)
% Difference (95% CI)	0.0 [Reference]	2.0 (–21.0 to 31.7)	–19.6 (–39.2 to 6.4)	–24.6 (–64.1 to 58.4)
CRP, median (IQR), mg/L	4.9 (2.7 to 9.4)	5.0 (2.7 to 9.1)	5.1 (2.1 to 8.0)	6.2 (5.6 to 6.3)
% Difference (95% CI)	0.0 [Reference]	–30.7 (–46.6 to –10.1)	–33.4 (–54.6 to –2.3)	–4.2 (–36.8 to 45.3)
Cholesterol, median (IQR), mg/dL	209.0 (170.0 to 233.0)	200.0 (177.0 to 223.0)	211.0 (180.0 to 241.0)	186.0 (185.0 to 211.0)
% Difference (95% CI)	0.0 [Reference]	–7.8 (–13.1 to –2.1)	–3.4 (–11.0 to 4.9)	–18.1 (–27.0 to –8.1)
Triglycerides, median (IQR), mg/dL	147.0 (119.0 to 180.0)	143.0 (109.0 to 187.0)	134.0 (101.0 to 158.0)	147.0 (141.0 to 155.0)
% Difference (95% CI)	0.0 [Reference]	–14.8 (–22.1 to –6.7)	–28.6 (–38.8 to –16.6)	–20.5 (–30.3 to –9.3)
HDL, median (IQR), mg/dL	69.0 (57.7 to 75.0)	68.8 (55.3 to 79.5)	76.0 (64.7 to 92.1)	70.0 (63.0 to 71.4)
% Difference (95% CI)	0.0 [Reference]	3.1 (–6.0 to 13.0)	24.4 (9.2 to 41.8)	1.8 (–16.4 to 24.0)
LDL, mg/dL	105.5 (75.4 to 126.1)	99.7 (81.3 to 121.2)	99.0 (83.1 to 135.8)	94.8 (88.8 to 95.7)
% Difference (95% CI)	0.0 [Reference]	–11.4 (–20.9 to –0.8)	–11.6 (–26.0 to 5.5)	–24.8 (–37.3 to –9.7)

Abbreviations: HDL, high-density lipoprotein; HOMA-IR, Homeostatic model assessment for insulin resistance; IQR, interquartile range; LDL, low-density lipoprotein.

To convert C-peptide to nmol/L, multiply by 0.331; HbA_1c_ to proportion of total hemoglobin, multiply by 0.01; glucose to mmol/L, multiply by 0.0555; CRP to mg/L, multiply by 10; cholesterol to mmol/L, multiply by 0.0259; triglycerides to mmol/L, multiply by 0.0113; HDL to mmol/L, multiply by 0.0259; LDL to mmol/L, multiply by 0.0259.

^a^ Analyses are weighted to be representative of the full cohort.

^b^ Continuous outcomes were log-transformed. Results are presented as the percent difference (95% CI) calculated as the exponentiated beta coefficient from the adjusted linear regression model with robust standard errors, subtracting 1 and multiplying by 100. Analyses adjusted for age, prepregnancy body mass index (calculated as weight in kilograms divided by height in meters squared), race and/or ethnicity, education, marital status, nulliparity, and gestational week at blood collection.

## Discussion

In this prospective cohort study, pregnant women who consumed low and moderate levels of caffeinated beverages early in the second trimester within current ACOG guidelines of less than 200 mg/d was associated with lower risk for GDM and lower glucose levels at GDM screening, compared with women who did not drink caffeinated beverages. These observations were substantiated by findings that second trimester caffeinated beverage intake was associated with lower CRP and C-peptide concentrations and more favorable lipid profiles. Additionally, increasing first trimester plasma caffeine and paraxanthine concentrations were associated with lower glucose concentrations at GDM screening. However, caffeine was not associated with gestational hypertension or preeclampsia risk nor blood pressure levels across pregnancy. These findings address critical data gaps on the health implications of caffeine consumption in pregnancy for maternal health.

Prior US and Danish cohort studies^12,13^ reported nonsignificant inverse associations between self-reported caffeinated coffee before and during the first trimester of pregnancy^12^ or the first trimester coffee and tea intake^13^ with GDM risk. The current study uniquely included second trimester measures and continuous measures of glucose and cardiometabolic markers. No significant association was observed with first trimester self-reported intake, but estimates for caffeinated beverage intake of less than 200 mg/d were consistent in magnitude, although imprecise. First trimester plasma caffeine and paraxanthine were associated with lower glucose levels on the GCT, but not GDM risk, although estimates were in a similar direction. This imprecision could be due to only a single observation in time or variations in caffeine metabolism,^34^ or it could be that associations are also driven by other beverage components besides caffeine. Coffee and tea contain phytochemicals that may affect inflammation and insulin resistance.^38^ Nevertheless, caffeine in itself has been associated with improved energy balance^39^ and decreased fat mass,^8^ which could play a role in the biological mechanism for the observed associations. Findings with reduced GDM risk are supported by improvements in fasting cardiometabolic profile. Additional research is needed to understand underlying molecular mechanisms for these findings and identify relevant components.

Neither low or moderate caffeinated beverage intake or plasma caffeine and paraxanthine were associated with risk for preeclampsia, gestational hypertension, or blood pressure across pregnancy. Very few women consumed high levels of caffeine, and thus it is difficult to make inferences about risk across the spectrum of potential intake. Null findings between caffeine and preeclampsia are somewhat surprising given that better lipid profiles were observed among women who drank caffeinated beverages in the second trimester and dyslipidemia has been associated with preeclampsia risk^40^ and could be due to a lack of statistical power. Prior studies on caffeine or coffee intake in pregnancy and gestational hypertension and preeclampsia risk have been inconsistent. One small study reported no association between second trimester paraxanthine and preecalmpsia.^15^ Another study found that mean intake across pregnancy of self-reported caffeinated coffee and tea was not associated with gestational hypertension or preeclampsia risk, although there was an inverse association between intakes of 180 mg/d to 351 mg/d and preeclampsia, which was reported as unexpected and needing replication.^14^ The latter study also observed a positive association between third trimester caffeine intake of 360 mg/d to 531 mg/d and systolic blood pressure.^14^ Further research about caffeine and gestational hypertension or preeclampsia risk in large cohorts are needed.

Currently ACOG recommends that pregnant women do not consume more than 200 mg/d of caffeine.^3^ This recommendation was based on findings that no associations for miscarriage, preterm delivery, or intrauterine growth restriction were observed at levels less than 200 mg/d, but that associations, although inconsistent, were observed between higher caffeine, miscarriage, and growth restriction. While this study could not make a determination for risks associated with caffeine consumption above current recommendations, findings of a lower GDM risk with low and moderate second trimester caffeine consumption do not conflict with current recommendations. Nevertheless, these findings are based on observational data, and therefore, residual confounding cannot be completely ruled out even though major known confounders were adjusted for in this study. Notably, recent systematic reviews^41,42^ concluded that there was no safe level of caffeine intake in pregnancy based on impacts on offspring outcomes. Similarly, we previously found that caffeine consumption during pregnancy, even in amounts less than the recommended 200 mg per day, was associated with smaller neonatal anthropometric measurements.^43^ Therefore, it would not be prudent for women do not drink caffeinated beverages to initiate consumption for the purpose of lowering GDM risk and improving glucose metabolism.

Strengths include prospective analysis based on a multicenter diverse cohort with comprehensive confounding factors and repeated assessments of caffeine intake. Availability of first trimester plasma caffeine and paraxanthine was also a strength as it avoids recall bias and also captures additional sources of caffeine such as decaffeinated coffee or chocolate. Additionally, associations with fasting cardiometabolic profiles were assessed to further understand etiology of findings.

### Limitations

The sample size was not large enough to separate analyses by beverage type and may have had limited power to detect associations due to small number of women with pregnancy complications. However, inclusion of continuous measures of glucose, blood pressure, and cardiometabolic markers addressed this limitation to some degree. Timing of diagnosis with gestational hypertension and preeclampsia was unknown, and therefore, whether caffeinated beverage intake later in pregnancy is associated with blood pressure, hypertension or preeclampsia risk requires future study. Also, the self-reported exposure represents caffeine from beverages only; notably only 2 women took medications containing caffeine.^44^

## Conclusions

In this cohort study, caffeinated beverage intake within the currently recommended range in the second trimester of pregnancy was associated with a lower GDM risk than with no intake, but not with preeclampsia or gestational hypertension. These findings based on self-reported information and biomarkers should be reassuring for pregnant women with moderate caffeine intake, but translation into public health recommendations should be considered in the context of published data on offspring outcomes.

## Notes

Supplement. **eTable 1.** Cardiometabolic Marker Assay Information **eTable 2.** Characteristics of Study Participants According to Plasma Total Caffeine and Paraxanthine Concentrations at 10 to 13 Weeks, NICHD Fetal Growth Studies-Singleton Cohort **eTable 3. **Sensitivity Analyses Assessing the Association of Self-reported Caffeine Intake and Risk for Either Gestational Diabetes or Impaired Glucose Tolerance **eTable 4.** Sensitivity Analyses With Additional Covariate Adjustment and Exclusion of Smokers for Self-reported Caffeine Intake and Associations With Gestational Diabetes and Glucose Challenge Test Results **eTable 5.** Sensitivity Analysis of Caffeine Metabolites at 10 to 13 Weeks Gestation and Associations With Gestational Diabetes or Impaired Glucose Tolerance **eTable 6.** Sensitivity Analyses With Additional Covariate Adjustment and Exclusion of Smokers for Analyses of Caffeine Metabolites at 10 to 13 Weeks Gestation and Associations With Gestational Diabetes and Glucose Challenge Test **eTable 7.** Primary Analyses and Sensitivity Analyses With Additional Covariates Adjustment and Exclusion of Smokers for Plasma Caffeine Metabolites at 10 to 13 Weeks Gestation and Associations With Gestational Hypertension and Preeclampsia **eTable 8.** Sensitivity Analyses With Additional Covariate Adjustment and Exclusion of Smokers for Analyses of Self-reported Caffeinated Beverage Intake and Associations With Gestational Hypertension and Preeclampsia **eFigure 1.** Sample Flow Chart **eFigure 2.** Prospective Adjusted Associations Between Self-reported Caffeinated Beverage Intake (mg/d) at 10 to 13 weeks and Total Plasma Caffeine and Paraxanthine at 10 to 13 Weeks Gestation and Blood Pressure Across Gestation

## References

[1] Fulgoni VL III, Keast DR, Lieberman HR. Trends in intake and sources of caffeine in the diets of US adults: 2001-2010. Am J Clin Nutr. 2015;101(5):1081-1087. doi:10.3945/ajcn.113.080077 25832334

[2] Chen L, Bell EM, Browne ML, Druschel CM, Romitti PA; National Birth Defects Prevention Study. Exploring maternal patterns of dietary caffeine consumption before conception and during pregnancy. Matern Child Health J. 2014;18(10):2446-2455. doi:10.1007/s10995-014-1483-2 24791972 PMC5901698

[3] ACOG Committee Opinion No. 462: Moderate caffeine consumption during pregnancy. Obstet Gynecol. 2010;116(2 Pt 1):467-468. doi:10.1097/AOG.0b013e3181eeb2a1 20664420

[4] van Dam RM, Hu FB, Willett WC. Coffee, caffeine, and health. N Engl J Med. 2020;383(4):369-378. doi:10.1056/NEJMra1816604 32706535

[5] De Giuseppe R, Di Napoli I, Granata F, Mottolese A, Cena H. Caffeine and blood pressure: a critical review perspective. Nutr Res Rev. 2019;32(2):169-175. doi:10.1017/S0954422419000015 30947761

[6] Grosso G, Micek A, Godos J, . Long-term coffee consumption is associated with decreased incidence of new-onset hypertension: a dose–response meta-analysis. Nutrients. 2017;9(8):890. doi:10.3390/nu9080890 28817085 PMC5579683

[7] Greer F, Hudson R, Ross R, Graham T. Caffeine ingestion decreases glucose disposal during a hyperinsulinemic-euglycemic clamp in sedentary humans. Diabetes. 2001;50(10):2349-2354. doi:10.2337/diabetes.50.10.2349 11574419

[8] Alperet DJ, Rebello SA, Khoo EY-H, . The effect of coffee consumption on insulin sensitivity and other biological risk factors for type 2 diabetes: a randomized placebo-controlled trial. Am J Clin Nutr. 2020;111(2):448-458. doi:10.1093/ajcn/nqz306 31891374

[9] van Dam RM, Willett WC, Manson JE, Hu FB. Coffee, caffeine, and risk of type 2 diabetes: a prospective cohort study in younger and middle-aged U.S. women. Diabetes Care. 2006;29(2):398-403. doi:10.2337/diacare.29.02.06.dc05-1512 16443894

[10] Sonagra AD, Biradar SM, K D, Murthy D S J. Normal pregnancy: a state of insulin resistance. J Clin Diagn Res. 2014;8(11):CC01-CC03. doi:10.7860/JCDR/2014/10068.5081 25584208 PMC4290225

[11] Knutti R, Rothweiler H, Schlatter C. The effect of pregnancy on the pharmacokinetics of caffeine. New Toxicology for Old. Springer; 1982:187-192. 10.1007/978-3-642-68511-8_33 6954898

[12] Adeney KL, Williams MA, Schiff MA, Qiu C, Sorensen TK. Coffee consumption and the risk of gestational diabetes mellitus. Acta Obstet Gynecol Scand. 2007;86(2):161-166. doi:10.1080/00016340600994992 17364278

[13] Hinkle SN, Laughon SK, Catov JM, Olsen J, Bech BH. First trimester coffee and tea intake and risk of gestational diabetes mellitus: a study within a national birth cohort. BJOG. 2015;122(3):420-428. doi:10.1111/1471-0528.12930 24947484 PMC4272679

[14] Bakker R, Steegers EA, Raat H, Hofman A, Jaddoe VW. Maternal caffeine intake, blood pressure, and the risk of hypertensive complications during pregnancy: the generation R study. Am J Hypertens. 2011;24(4):421-428. doi:10.1038/ajh.2010.242 21164492

[15] Eichelberger KY, Baker AM, Woodham PC, Haeri S, Strauss RA, Stuebe AM. Second-trimester maternal serum paraxanthine, CYP1A2 activity, and the risk of severe preeclampsia. Obstet Gynecol. 2015;126(4):725-730. doi:10.1097/AOG.0000000000001041 26348183

[16] Grewal J, Grantz KL, Zhang C, . Cohort profile: NICHD fetal growth studies–singletons and twins. Int J Epidemiol. 2018;47(1):25-25l. doi:10.1093/ije/dyx161 29025016 PMC5837516

[17] Buck Louis GM, Grewal J, Albert PS, . Racial/ethnic standards for fetal growth: the NICHD fetal growth studies. Am J Obstet Gyncol. 2015;213(4):449 e1-449 e41. doi:10.1016/j.ajog.2015.08.032 PMC4584427 26410205

[18] Zhu Y, Mendola P, Albert PS, . Insulin-like growth factor axis and gestational diabetes mellitus: a longitudinal study in a multiracial cohort. Diabetes. 2016;65(11):3495-3504. doi:10.2337/db16-0514 27468747 PMC5079637

[19] Service. USDoAAR. FoodData Central. Accessed October 20, 2021. https://fdc.nal.usda.gov/

[20] Honda M, Robinson M, Kannan K. A rapid method for the analysis of perfluorinated alkyl substances in serum by hybrid solid-phase extraction. Environmental Chemistry. 2018;15(2):92-99. doi:10.1071/EN17192

[21] Richardson DB, Ciampi A. Effects of exposure measurement error when an exposure variable is constrained by a lower limit. Am J Epidemiol. 2003;157(4):355-363. doi:10.1093/aje/kwf217 12578806

[22] Schisterman EF, Vexler A, Whitcomb BW, Liu A. The limitations due to exposure detection limits for regression models. Am J Epidemiol. 2006;163(4):374-383. doi:10.1093/aje/kwj039 16394206 PMC1408541

[23] Mateus J, Newman RB, Zhang C, Fetal growth patterns in pregnancy-associated hypertensive disorders: NICHD fetal growth studies. Am J Obstet Gynecol. 2019;221(6):635. 31226296 10.1016/j.ajog.2019.06.028 PMC6888945

[24] ACOG practice bulletin No. 190: gestational diabetes mellitus. Am J Obstet Gynecol. 2018;131(2):e49-e64. doi:10.1097/AOG.0000000000002501 29370047

[25] American Diabetes Association. 2. Classification and diagnosis of diabetes. Diabetes Care. 2017;40(suppl 1):S11-S24. doi:10.2337/dc17-S005 27979889

[26] Wallace TM, Levy JC, Matthews DR. Use and abuse of HOMA modeling. Diabetes Care. 2004;27(6):1487-1495. doi:10.2337/diacare.27.6.1487 15161807

[27] Boyko EJ, Jensen CC. Do we know what homeostasis model assessment measures? If not, does it matter? Diabetes Care. 2007;30(10):2725-2728. doi:10.2337/dc07-1248 17901529

[28] Chasan-Taber L, Schmidt MD, Roberts DE, Hosmer D, Markenson G, Freedson PS. Development and validation of a pregnancy physical activity questionnaire. Med Sci Sports Exerc. 2004;36(10):1750-1760. doi:10.1249/01.MSS.0000142303.49306.0D 15595297

[29] Cohen S, Kamarck T, Mermelstein R. A global measure of perceived stress. J Health Soc Behav. 1983;24(4):385-396. doi:10.2307/2136404 6668417

[30] Hinkle SN, Zhang C, Grantz KL, Nutrition during pregnancy: findings from the National Institute of Child Health and Human Development (NICHD) Fetal Growth Studies–Singleton Cohort. Curr Dev Nutr. 2021;5(1):nzaa182. doi:10.1093/cdn/nzaa182 33553996 PMC7846139

[31] Guenther PM, Casavale KO, Reedy J, . Update of the healthy eating index: HEI-2010. J Acad Nutr Diet. 2013;113(4):569-580. doi:10.1016/j.jand.2012.12.016 23415502 PMC3810369

[32] Zou G. A modified Poisson regression approach to prospective studies with binary data. Am J Epidemiol. 2004;159(7):702-706. doi:10.1093/aje/kwh090 15033648

[33] Kleinbaum DG, Klein M. Logistic Regression: A Self-Learning Text. Springer New York; 2010. doi:10.1007/978-1-4419-1742-3

[34] Grosso LM, Bracken MB. Caffeine metabolism, genetics, and perinatal outcomes: a review of exposure assessment considerations during pregnancy. Ann Epidemiol. 2005;15(6):460-466. doi:10.1016/j.annepidem.2004.12.011 15967394

[35] Zhou XH, Eckert GJ, Tierney WM. Multiple imputation in public health research. Stat Med. 2001;20(9-10):1541-1549. doi:10.1002/sim.689 11343373

[36] Samuelsen SO. A psudolikelihood approach to analysis of nested case-control studies. Biometrika. 1997;84(2):379-394. doi:10.1093/biomet/84.2.379

[37] Hinkle SN, Rawal S, Liu D, Chen J, Tsai MY, Zhang C. Maternal adipokines longitudinally measured across pregnancy and their associations with neonatal size, length, and adiposity. Int J Obesity. 2019;43(7):1422-1434. Medline:30464233. 30464233 10.1038/s41366-018-0255-2 PMC6529296

[38] Rebollo-Hernanz M, Zhang Q, Aguilera Y, Martín-Cabrejas MA, Gonzalez de Mejia E. Phenolic compounds from coffee by-products modulate adipogenesis-related inflammation, mitochondrial dysfunction, and insulin resistance in adipocytes, via insulin/PI3K/AKT signaling pathways. Food Chem Toxicol. 2019;132:110672. doi:10.1016/j.fct.2019.110672 31306686

[39] Harpaz E, Tamir S, Weinstein A, Weinstein Y. The effect of caffeine on energy balance. J Basic Clin Physiol Pharmacol. 2017;28(1):1-10. doi:10.1515/jbcpp-2016-0090 27824614

[40] Spracklen CN, Smith CJ, Saftlas AF, Robinson JG, Ryckman KK. Maternal hyperlipidemia and the risk of preeclampsia: a meta-analysis. Am J Epidemiol. 2014;180(4):346-358. doi:10.1093/aje/kwu145 24989239 PMC4565654

[41] James JE. Maternal caffeine consumption and pregnancy outcomes: a narrative review with implications for advice to mothers and mothers-to-be. BMJ evidence-based medicine. 2020;26(3):114-115. 32843532 10.1136/bmjebm-2020-111432 PMC8165152

[42] Jin F, Qiao C. Association of maternal caffeine intake during pregnancy with low birth weight, childhood overweight, and obesity: a meta-analysis of cohort studies. International Journal of Obesity. 2020;45(2):279-287. 32518355 10.1038/s41366-020-0617-4

[43] Gleason JL, Tekola-Ayele F, Sundaram R, . Association between maternal caffeine consumption and metabolism and neonatal anthropometry: a secondary analysis of the NICHD fetal growth studies-singletons. JAMA Netw Open. 2021;4(3):e213238. doi:10.1001/jamanetworkopen.2021.3238 33764424 PMC7994948

[44] Vafai Y, Yeung EH, Sundaram R, . Prenatal medication use in a prospective pregnancy cohort by pre-pregnancy obesity status. J Matern Fetal Neonatal Med. 2021;1-8. doi:10.1080/14767058.2021.1893296 33706661 PMC8802334

