# Supplementary material for: Assessment of Caffeine Consumption and Maternal Cardiometabolic Pregnancy Complications
Source: JAMA Netw Open. 2021 Nov 8;4(11):e2133401. doi: 10.1001/jamanetworkopen.2021.33401 (PMC8576579; doi:10.1001/jamanetworkopen.2021.33401)
Supplement: Supplement. — eTable 1. Cardiometabolic Marker Assay Information eTable 2. Characteristics of Study Participants According to Plasma Total Caffeine and Paraxanthine Concentrations at 10 to 13 Weeks, NICHD Fetal Growth Studies-Singleton Cohort eTable 3. Sensitivity Analyses Assessing the Association of Self-reported Caffeine Intake and Risk for Either Gestational Diabetes or Impaired Glucose Tolerance eTable 4. Sensitivity Analyses With Additional Covariate Adjustment and Exclusion of Smokers for Self-reported Caffeine Intake and Associations With Gestational Diabetes and Glucose Challenge Test Results eTable 5. Sensitivity Analysis of Caffeine Metabolites at 10 to 13 Weeks Gestation and Associations With Gestational Diabetes or Impaired Glucose Tolerance eTable 6. Sensitivity Analyses With Additional Covariate Adjustment and Exclusion of Smokers for Analyses of Caffeine Metabolites at 10 to 13 Weeks Gestation and Associations With Gestational Diabetes and Glucose Challenge Test eTable 7. Primary Analyses and Sensitivity Analyses With Additional Covariates Adjustment and Exclusion of Smokers for Plasma Caffeine Metabolites at 10 to 13 Weeks Gestation and Associations With Gestational Hypertension and Preeclampsia eTable 8. Sensitivity Analyses With Additional Covariate Adjustment and Exclusion of Smokers for Analyses of Self-reported Caffeinated Beverage Intake and Associations With Gestational Hypertension and Preeclampsia eFigure 1. Sample Flow Chart eFigure 2. Prospective Adjusted Associations Between Self-reported Caffeinated Beverage Intake (mg/d) at 10 to 13 weeks and Total Plasma Caffeine and Paraxanthine at 10 to 13 Weeks Gestation and Blood Pressure Across Gestation [file jamanetwopen-e2133401-s001.pdf]

Supplemental Online Content

Hinkle SN, Gleason JL, Yisahak SF, et al. Assessment of caffeine consumption and maternal cardiometabolic pregnancy complications. *JAMA Netw Open.* 2021;4(11):e2133401. doi:10.1001/jamanetworkopen.2021.33401

eTable 1. Cardiometabolic Marker Assay Information

eTable 2. Characteristics of Study Participants According to Plasma Total Caffeine and Paraxanthine Concentrations at 10 to 13 Weeks, NICHD Fetal Growth Studies-Singleton Cohort

eTable 3. Sensitivity Analyses Assessing the Association of Self-reported Caffeine Intake and Risk for Either Gestational Diabetes or Impaired Glucose Tolerance

eTable 4. Sensitivity Analyses With Additional Covariate Adjustment and Exclusion of Smokers for Self-reported Caffeine Intake and Associations With Gestational Diabetes and Glucose Challenge Test Results

eTable 5. Sensitivity Analysis of Caffeine Metabolites at 10 to 13 Weeks Gestation and Associations With Gestational Diabetes or Impaired Glucose Tolerance

eTable 6. Sensitivity Analyses With Additional Covariate Adjustment and Exclusion of Smokers for Analyses of Caffeine Metabolites at 10 to 13 Weeks Gestation and Associations With Gestational Diabetes and Glucose Challenge Test

eTable 7. Primary Analyses and Sensitivity Analyses With Additional Covariates Adjustment and Exclusion of Smokers for Plasma Caffeine Metabolites at 10 to 13 Weeks Gestation and Associations With Gestational Hypertension and Preeclampsia

eTable 8. Sensitivity Analyses With Additional Covariate Adjustment and Exclusion of Smokers for Analyses of Self-reported Caffeinated Beverage Intake and Associations With Gestational Hypertension and Preeclampsia

eFigure 1. Sample Flow Chart

eFigure 2. Prospective Adjusted Associations Between Self-reported Caffeinated Beverage Intake (mg/d) at 10 to 13 weeks and Total Plasma Caffeine and Paraxanthine at 10 to 13 Weeks Gestation and Blood Pressure Across Gestation

This supplemental material has been provided by the authors to give readers additional information about their work.

© 2021 Hinkle SN et al. *JAMA Network Open.*

eTable 1. Cardiometabolic Marker Assay Information

Biomarker	Units	Kit	Manufacturer
Cholesterol	mg/dL	Roche COBAS 6000 Chemistry Analyzer	Roche Diagnostics
C-Peptide	nmol/L	Roche Elecsys 2010 Analyzer using a sandwich immunoassay method	Roche Diagnostics
Glucose	md/dL	Enzymatic assays with the use of the Roche Modular P Chemistry analyzer	Roche Diagnostics
HbA1c	%	Non-porous ion Exchange High Performance Liquid Chromatography	Tosoh Bioscience, Inc
HDLD	mg/dL	Roche COBAS 6000 Chemistry Analyzer	Roche Diagnostics
hsCRP	mg/L	enzymatic assays with the use of the Roche Modular P Chemistry analyzer	Roche Diagnostics
Insulin	pmol/L	Roche Elecsys 2010 Analyzer	Roche Diagnostics
LDLD	mg/dL	calculated as = total cholesterol − HDL cholesterol − triglycerides ÷ 5	
Triglycerides	mg/dL	Roche COBAS 6000 Chemistry Analyzer	Roche Diagnostics

Abbreviations: hsCRP, high sensitivity C-reactive protein; HDL, high-density lipoprotein; LDL, low-density lipoprotein.
All inter and intra assay CVs <4.3%.

© 2021 Hinkle SN et al. *JAMA Network Open.*

eTable 2. Characteristics of Study Participants According to Plasma Total Caffeine and Paraxanthine Concentrations at 10 to 13 Weeks, NICHD Fetal Growth Studies-Singleton Cohort

	Mean (SD), Median (Interquartile Range), or n (%)					
	Plasma Total Caffeine and Paraxanthine					
	Overall	Quartile 1[a]	Quartile 2[a]	Quartile 3[a]	Quartile 4[a]	
Characteristics[b]	(n=2529)	(n=608)	(n=608)	(n=608)	(n=607)	*P*[c]
Age, years	28.2 (5.5)	27.5 (5.5)	28.3 (5.5)	27.5 (5.4)	29.4 (5.3)	<0.001
Race/ethnicity						<0.001
Asian/Pacific Islander	412 (16%)	134 (22%)	94 (15%)	71 (12%)	102 (17%)	
Hispanic	730 (29%)	135 (22%)	184 (30%)	221 (36%)	157 (26%)	
Non-Hispanic Black	685 (27%)	235 (39%)	166 (27%)	148 (24%)	111 (18%)	
Non-Hispanic White	702 (28%)	104 (17%)	164 (27%)	168 (28%)	237 (39%)	
Pre-pregnancy BMI, kg/m^2	25.5 (5.2)	25.1 (5.0)	25.1 (4.9)	25.7 (5.0)	25.2 (5.1)	0.05
Nulliparous	1182 (47%)	341 (56%)	308 (51%)	265 (44%)	220 (36%)	<0.001
Education						0.001
≤ High school	749 (30%)	203 (33%)	168 (28%)	194 (32%)	151 (25%)	
Some college/ associate degree	760 (30%)	182 (30%)	173 (28%)	194 (32%)	175 (29%)	
≥ 4-year college degree	1020 (40%)	223 (37%)	267 (44%)	220 (36%)	281 (46%)	
Married	1789 (71%)	434 (71%)	431 (71%)	426 (70%)	433 (71%)	0.96
Fulltime job or student	1891 (75%)	407 (67%)	469 (77%)	455 (75%)	488 (81%)	<0.001
Family history of diabetes	530 (22%)	120 (21%)	129 (22%)	128 (22%)	121 (21%)	0.92
Severe first trimester vomiting or hyperemesis	123 (5%)	26 (4%)	30 (5%)	32 (5%)	29 (5%)	0.88
Alcohol intake prior to pregnancy, number of drinks per day	0.0 (0.0, 4.3)	0.0 (0.0, 0.1)	0.0 (0.0, 4.3)	0.0 (0.0, 4.3)	0.1 (0.0, 4.3)	<0.001
Moderate and vigorous physical activity, past year, MET HR/wk	96.7 (51.6, 169.5)	94.4 (48, 162.4)	90.3 (49.4, 168.2)	93.5 (50.9, 155.1)	105.5 (59.6, 181.7)	0.007
Perceived stress score	11.5 (6.2)	11.7 (6.0)	11.1 (6.2)	11.9 (6.4)	11.2 (6.4)	0.05
Sleep, hours	8.1 (1.5)	8.2 (1.5)	8.2 (1.4)	8.1 (1.5)	8.1 (1.4)	0.25
Cotinine, ng/mL	0.0 (0.0, 0.1)	0.0 (0.0, 0.1)	0.0 (0.0, 0.0)	0.0 (0.0, 0.1)	0.0 (0.0, 0.1)	0.25
Total energy, past three months, kcal/d	1904 (1432, 2607)	2022 (1463, 2767)	1858 (1429, 2503)	1862 (1424, 2609)	1850 (1430, 2544)	0.39
Healthy Eating Index-2010 total score	65.1 (10.3)	64.7 (10.2)	65.8 (10.5)	64.6 (10.4)	65.3 (9.9)	0.31

Abbreviations: BMI, body mass index; MET, metabolic equivalent of task; SD, standard deviation.

[a] The range for total plasma caffeine and paraxanthine in each quartile was: -5.0-48.7; 48.8-243.0; 243.3-903.6; 905.7-13269.8 µg/dL

[b] Missing: pre-pregnancy BMI n=18; employment n=1; marital status n=2; family history of diabetes n=79; alcohol intake n=1; physical activity n=6; stress n=23; sleep n=3; cotinine n=98; total energy n=1017; Healthy Eating Index-2010 total score n=1017; caffeine and paraxanthine n=98.

© 2021 Hinkle SN et al. *JAMA Network Open.*

[c] Differences in continuous covariates was assessed using a one-way analysis of variance (ANOVA) for normally distributed continuous variables, the Kruskal Wallis test for non-parametric continuous variables, and chi square test for categorical variables.

© 2021 Hinkle SN et al. *JAMA Network Open.*

eTable 3. Sensitivity Analyses Assessing the Association of Self-reported Caffeine Intake and Risk for Either Gestational Diabetes or Impaired Glucose Tolerance

	Relative Risk for Gestational Diabetes or Impaired Glucose Tolerance (95% CI)[a]				
	Past Week Caffeinated Beverage Intake, mg/d				
	**0**	**1-100**	**101-200**	**>200**	**Per 50**
**10-13 weeks**					
% Outcome	9.9%	8.4%	10.3%	16.7%	
Unadjusted	1.00 (Reference)	0.85 (0.65,1.10)	1.00 (0.61,1.66)	1.68 (0.59,4.81)	1.03 (0.86,1.22)
Adjusted[b]	1.00 (Reference)	0.80 (0.62,1.03)	0.95 (0.58,1.56)	2.58 (0.80,8.33)	1.01(0.81,1.27)
**16-22 weeks**					
% Outcome	11.6%	8.5%	7.9%	4.9%	
Unadjusted	1.00 (Reference)	0.71 (0.53,0.94)	0.64 (0.36,1.14)	0.45 (0.07,3.07)	0.84 (0.71,0.99)
Adjusted[b]	1.00 (Reference)	0.66 (0.50,0.88)	0.55 (0.31,0.98)	0.55 (0.07,4.03)	0.78 (0.66,0.93)

[a] Relative risks estimated using a log Poisson model with robust variance with covariates multiply imputed (M=20).
[b] Models adjusted for age, pre-pregnancy BMI, race/ethnicity, education, marital status, and nulliparity,

© 2021 Hinkle SN et al. *JAMA Network Open.*

eTable 4. Sensitivity Analyses With Additional Covariate Adjustment and Exclusion of Smokers for Self-reported Caffeine Intake and Associations With Gestational Diabetes and Glucose Challenge Test Results

	**Relative Risk for Gestational Diabetes (95% CI)[a]**					**Glucose Challenge Test Results, mg/dL (95% CI)[b]**				
	**Past Week Caffeinated Beverage Intake, mg/d**					**Past Week Caffeinated Beverage Intake, mg/d**				
	**0**	**1-100**	**101-200**	**>200**	**Per 50**	**0**	**1-100**	**101-200**	**>200**	**Per 50**
**10-13 weeks**										
Fully Adjusted[c]	1.00 (Reference)	0.70 (0.47,1.05)	0.91 (0.42,1.95)	2.00 (0.26,15.53	0.96 (0.76,1.22)	0.0 (Reference)	-2.0 (-4.3,0.3)	-0.4 (-5.0,4.2)	1.1 (-11.5,13.7)	-0.3 (-1.4,0.9)
Among non-smokers only[d]	1.00 (Reference	0.72 (0.48,1.07)	1.01 (0.49,2.09)	1.96 (0.25,15.34	1.00 (0.80,1.25)	0.0 (Reference	-1.7 (-4.0,0.6)	0.2 (-4.4,4.8)	1.2 (-11.3,13.8)	-0.1 (-1.3,1.0)
**16-22 weeks**										
Fully Adjusted[f]	1.00 (Reference)	0.52 (0.34,0.79)	0.50 (0.22,1.11)	--[e]	0.73 (0.55,0.96)	0.0 (Reference)	-2.5 (-5.3,0.2)	-4.7 (-9.4,0.1)	-1.8 (-14.0,10.4)	-1.1 (-2.3,0.1)
Among non-smokers only[d]	1.00 (Reference	0.51 (0.33,0.78)	0.56 (0.26,1.23)	--[e]	0.74 (0.55,0.98)	0.0 (Reference	-2.7 (-5.4,0.0)	-4.4 (-9.1,0.3)	-1.3 (-13.6,10.9)	-1.1 (-2.3,0.1)

[a] Relative risks estimated using a log Poisson model with robust variance with covariates multiply imputed (M=20).
[b] Continuous glucose challenge test results estimated using linear regression model with covariates and outcome multiply imputed (M=20).
[c] Models adjusted for a priori covariates of age, pre-pregnancy BMI, race/ethnicity, education, marital status, and nulliparity plus pre-pregnancy alcohol, perceived stress at 10-13 weeks, plasma cotinine at 10-13 weeks, periconception and first trimester Healthy Eating Index-2010, and family history of diabetes.
[d] Models adjusted for age, pre-pregnancy BMI, race/ethnicity, education, marital status, and nulliparity,
[e] Women with caffeine intake >200 excluded due to lack of model convergence from a small sample size and no cases of GDM within this category.
[f] Models adjusted for a priori covariates of age, pre-pregnancy BMI, race/ethnicity, education, marital status, and nulliparity plus, perceived stress at 16-22 weeks, moderate and vigorous physical activity since enrollment, periconception and first trimester Healthy Eating Index-2010, and family history of diabetes.

© 2021 Hinkle SN et al. *JAMA Network Open.*

eTable 5. Sensitivity Analysis of Caffeine Metabolites at 10 to 13 Weeks Gestation and Associations With Gestational Diabetes or Impaired Glucose Tolerance

	Relative Risk for Gestational Diabetes or Impaired Glucose Tolerance (95% CI)[a,b]					
	**Quartile 1**	**Quartile 2**	**Quartile 3**	**Quartile 4**	**P-Trend**	**Per 100 ug/L**
**Caffeine**						
% Outcome	8.7%	8.7%	10.4%	8.8%		
Unadjusted	1.00 (Reference)	1.00 (0.67,1.51)	1.15 (0.78,1.70)	1.01 (0.69,1.49)	0.88	0.99 (0.98,1.01)
Adjusted	1.00 (Reference)	1.01 (0.68,1.51)	1.09 (0.75,1.60)	0.90 (0.61,1.34)	0.41	0.99 (0.98,1.01)
**Paraxanthine**						
% Outcome	8.3%	9.5%	10.7%	8.2%		
Unadjusted	1.00 (Reference)	1.13 (0.78,1.64)	1.25 (0.86,1.82)	0.97 (0.65,1.45)	0.54	0.98 (0.94,1.03)
Adjusted	1.00 (Reference)	1.12 (0.78,1.61)	1.17 (0.81,1.69)	0.84 (0.56,1.26)	0.16	0.96 (0.92,1.01)
**Total Caffeine + Paraxanthine**						
% Outcome	8.3%	9.8%	9.5%	9.1%		
Unadjusted	1.00 (Reference)	1.16 (0.80,1.68)	1.10 (0.74,1.64)	1.09 (0.75,1.59)	0.99	1.00 (0.98,1.01)
Adjusted	1.00 (Reference)	1.14 (0.79,1.63)	1.06 (0.72,1.56)	0.96 (0.65,1.40)	0.47	0.99 (0.98,1.00)

[a] Relative risks estimated using a log Poisson model with robust variance with exposure and covariates multiply imputed (M=20).
[b] Models adjusted for age, pre-pregnancy BMI, race/ethnicity, education, marital status, and nulliparity.

© 2021 Hinkle SN et al. *JAMA Network Open.*

eTable 6. Sensitivity Analyses With Additional Covariate Adjustment and Exclusion of Smokers for Analyses of Caffeine Metabolites at 10 to 13 Weeks Gestation and Associations With Gestational Diabetes and Glucose Challenge Test

	Relative Risk for Gestational Diabetes (95% CI)[a]						Glucose Challenge Test Results, mg/dL (95% CI)[b]					
	**Quartile 1**	**Quartile 2**	**Quartile 3**	**Quartile 4**	**P-Trend**	**Per 100 ug/dL**	**Quartile 1**	**Quartile 2**	**Quartile 3**	**Quartile 4**	**P-Trend**	**Per 100 ug/dL**
**Plasma Caffeine**												
Fully Adjusted[c]	1.0 (Reference)	0.86 (0.47,1.59)	0.93 (0.54,1.61)	0.81 (0.44,1.49)	0.58	0.98 (0.96,1.01)	0.0 (Reference	-1.4 (-4.8,1.9)	-0.4 (-3.7,2.9)	-3.7 (-7.0,-0.4)	0.02	-0.1 (-0.2,0.0)
Among non-smokers only[d]	1.0 (Reference)	0.87 (0.46,1.63)	0.96 (0.54,1.68)	0.87 (0.48,1.60)	0.78	0.99 (0.96,1.01)	0.0 (Reference)	-1.3 (-4.7,2.0)	-0.3 (-3.6,3.0)	-3.5 (-6.9,-0.2)	0.03	-0.1 (-0.2,0.0)
**Plasma Paraxanthine**												
Fully Adjusted[c]	1.0 (Reference)	0.92 (0.52,1.61)	1.08 (0.61,1.89)	0.86 (0.47,1.58)	0.68	0.97 (0.93,1.03)	0.0 (Reference	-1.2 (-4.3,2.0)	0.9 (-2.5,4.2)	-4.3 (-7.6,-0.9)	0.004	-0.6 (-0.9,-0.2)
Among non-smokers only[d]	1.0 (Reference)	0.92 (0.51,1.67)	1.14 (0.65,1.99)	0.92 (0.49,1.71)	0.86	0.97 (0.91,1.04)	0.0 (Reference)	-1.0 (-4.2,2.2)	1.1 (-2.3,4.4)	-4.3 (-7.6,-0.9)	0.004	-0.6 (-0.9,-0.2)
**Plasma Total Caffeine + Paraxanthine**												
Fully Adjusted[c]	1.0 (Reference)	0.92 (0.52,1.62)	1.00 (0.57,1.75)	0.84 (0.46,1.52)	0.59	0.99 (0.97,1.01)	0.0 (Reference	-0.9 (-4.1,2.4)	-0.5 (-3.9,2.9)	-3.7 (-6.9,-0.4)	0.02	-0.1 (-0.2,0.0)
Among non-smokers only[d]	1.0 (Reference)	0.91 (0.51,1.66)	1.03 (0.58,1.82)	0.89 (0.49,1.61)	0.75	0.99 (0.97,1.01)	0.0 (Reference)	-0.7 (-3.9,2.5)	-0.3 (-3.7,3.0)	-3.7 (-7.0,-0.5)	0.01	-0.1 (-0.2,0.0)

[a] Relative risks estimated using a log Poisson model with robust variance with missing exposure and covariates multiply imputed (M=20).
[b] Continuous glucose challenge test results estimated using linear regression model with exposure, covariates, and outcome multiply imputed (M=20).
[c] Models adjusted for a priori covariates of age, pre-pregnancy BMI, race/ethnicity, education, marital status, and nulliparity plus pre-pregnancy alcohol, past year moderate and vigorous physical activity, and family history of diabetes.
[d] Models adjusted for a priori covariates of age, pre-pregnancy BMI, race/ethnicity, education, marital status, and nulliparity.

© 2021 Hinkle SN et al. *JAMA Network Open.*

eTable 7. Primary Analyses and Sensitivity Analyses With Additional Covariates Adjustment and Exclusion of Smokers for Plasma Caffeine Metabolites at 10 to 13 Weeks Gestation and Associations With Gestational Hypertension and Preeclampsia

	Odds Ratio for Gestational Hypertension (95% CI)[a]						Odds Ratio for Preeclampsia (95% CI)[a]					
**Plasma Caffeine**	**Quartile 1**	**Quartile 2**	**Quartile 3**	**Quartile 4**	**P-Trend**	**Per 100 ug/dl**	**Quartile 1**	**Quartile 2**	**Quartile 3**	**Quartile 4**	**P-Trend**	**Per 100 ug/dl**
Outcome %	4.1%	2.4%	3.8%	2.7%			3.2%	4.2%	4.9%	2.6%		
Unadjusted	1.00 (Reference)	0.58 (0.29,1.17)	0.94 (0.51,1.74)	0.64 (0.33,1.24)	0.38	0.98 (0.95,1.01)	1.00 (Reference)	1.19 (0.65,2.19)	1.52 (0.85,2.72)	0.76 (0.38,1.53)	0.17	0.99 (0.97,1.02)
Adjusted[b]	1.00 (Reference)	0.67 (0.33,1.38)	1.02 (0.54,1.93)	0.76 (0.37,1.53)	0.62	0.99 (0.96,1.02)	1.00 (Reference)	1.46 (0.78,2.74)	1.77 (0.97,3.22)	1.07 (0.52,2.19)	0.57	1.01 (0.99,1.03)
Fully Adjusted[c]	1.00 (Reference)	0.67 (0.33,1.39)	1.02 (0.54,1.92)	0.75 (0.37,1.51)	0.59	0.99 (0.96,1.02)	1.00 (Reference)	1.49 (0.79,2.81)	1.80 (0.98,3.29)	1.07 (0.52,2.22)	0.56	1.01 (0.99,1.03)
Among non-smokers only[b]	1.00 (Reference)	0.67 (0.32,1.37)	1.01 (0.54,1.91)	0.76 (0.38,1.54)	0.64	0.99 (0.96,1.02)	1.00 (Reference)	1.46 (0.78,2.74)	1.75 (0.96,3.20)	1.08 (0.52,2.22)	0.60	1.01 (0.99,1.03)
**Plasma Paraxanthine**												
Outcome %	3.8%	2.9%	3.8%	2.7%			3.4%	4.3%	3.79%	3.4%		
Unadjusted	1.00 (Reference)	0.78 (0.40,1.51)	1.01 (0.55,1.86)	0.70 (0.35,1.38)	0.38	1.02 (0.97,1.08)	1.00 (Reference)	1.27 (0.70,2.32)	1.09 (0.59,2.02)	1.00 (0.53,1.89)	0.68	0.98 (0.90,1.06)
Adjusted[b]	1.00 (Reference)	0.83 (0.42,1.63)	1.10 (0.58,2.08)	0.71 (0.34,1.44)	0.36	1.01 (0.95,1.06)	1.00 (Reference)	1.44 (0.78,2.66)	1.27 (0.67,2.41)	1.21 (0.62,2.35)	0.97	0.98 (0.92,1.05)
Fully Adjusted[c]	1.00 (Reference)	0.82 (0.42,1.62)	1.10 (0.58,2.08)	0.69 (0.34,1.42)	0.33	1.01 (0.95,1.06)	1.00 (Reference)	1.46 (0.79,2.72)	1.28 (0.67,2.44)	1.21 (0.62,2.36)	0.99	0.98 (0.91,1.05)
Among non-	1.00 (Reference)	0.82 (0.42,1.61)	1.09 (0.58,2.07)	0.71 (0.35,1.46)	0.39	1.01 (0.96,1.06)	1.00 (Reference)	1.43 (0.77,2.64)	1.27 (0.67,2.40)	1.22 (0.63,2.37)	0.93	0.98 (0.92,1.05)

© 2021 Hinkle SN et al. *JAMA Network Open.*

smokers only[b]												
**Plasma Total Caffeine + Paraxanthine**												
Outcome %	4.1%	2.3%	3.9%	2.9%			3.2%	4.2%	4.2%	3.2%		
Unadjusted	1.00 (Reference)	0.54 (0.26,1.09)	0.94 (0.52,1.71)	0.69 (0.36,1.30)	0.58	0.99 (0.97,1.01)	1.00 (Reference)	1.32 (0.72,2.42)	1.34 (0.73,2.47)	0.99 (0.52,1.91)	0.55	0.99 (0.98,1.01)
Adjusted[b]	1.00 (Reference)	0.57 (0.27,1.17)	1.02 (0.55,1.91)	0.77 (0.39,1.52)	0.81	1.00 (0.97,1.02)	1.00 (Reference)	1.52 (0.81,2.84)	1.56 (0.83,2.94)	1.35 (0.68,2.67)	0.83	1.00 (0.98,1.02)
Fully Adjusted[c]	1.00 (Reference)	0.57 (0.28,1.18)	1.02 (0.54,1.91)	0.76 (0.38,1.49)	0.76	1.00 (0.97,1.02)	1.00 (Reference)	1.54 (0.82,2.88)	1.61 (0.86,3.05)	1.34 (0.67,2.66)	0.88	1.00 (0.98,1.02)
Among non-smokers only[b]	1.00 (Reference)	0.56 (0.27,1.16)	1.02 (0.55,1.90)	0.79 (0.40,1.54)	0.87	1.00 (0.97,1.02)	1.00 (Reference)	1.51 (0.81,2.83)	1.56 (0.83,2.93)	1.37 (0.69,2.71)	0.78	1.00 (0.99,1.02)

[a] Odds ratios estimated using multinomial logistic regression models with exposures and covariates multiply imputed (M=20).
[b] Models adjusted for a priori covariates of age, pre-pregnancy BMI, race/ethnicity, education, marital status, and nulliparity.
[c] Models adjusted for a priori covariates of age, pre-pregnancy BMI, race/ethnicity, education, marital status, and nulliparity plus pre-pregnancy alcohol intake, perceived stress at 10-13 weeks, plasma cotinine at 10-13 weeks, periconception and first trimester Healthy Eating Index-2010, past year moderate and vigorous physical activity.

© 2021 Hinkle SN et al. *JAMA Network Open.*

eTable 8. Sensitivity Analyses With Additional Covariate Adjustment and Exclusion of Smokers for Analyses of Self-reported Caffeinated Beverage Intake and Associations With Gestational Hypertension and Preeclampsia

	Odds Ratio for Gestational Hypertension (95% CI)[a]					Odds Ratio for Preeclampsia (95% CI)[a]				
**Caffeinated Beverages**	**0 mg/d**	**1-100 mg/d**	**101-200 mg/d**	**>200 mg/d**	**Per 50 mg/d**	**0 mg/d**	**1-100 mg/d**	**101-200 mg/d**	**>200 mg/d**	**Per 50 mg/d**
**Past week intake, 10-13 weeks**										
Fully Adjusted[b]	1.00 (Reference)	0.94 (0.58,1.52)	0.91 (0.36,2.28)	1.35 (0.16,11.40)	0.98 (0.77,1.26)	1.00 (Reference)	1.21 (0.77,1.90)	0.81 (0.30,2.15)	--[c]	0.97 (0.77,1.23)
Among non-smokers only[d]	1.00 (Reference)	0.94 (0.58,1.52)	0.96 (0.38,2.39)	1.37 (0.16,11.41)	1.00 (0.78,1.27)	1.00 (Reference)	1.20 (0.77,1.87)	0.81 (0.31,2.13)	--[c]	0.97 (0.77,1.23)
**Past week intake, 16-22 weeks**										
Fully Adjusted[b]	1.00 (Reference)	1.14 (0.65,1.99)	0.97 (0.36,2.61)	--[c]	0.89 (0.67,1.16)	1.00 (Reference)	0.98 (0.59,1.64)	1.21 (0.51,2.87)	2.92 (0.60,14.22)	1.15 (0.94,1.42)
Among non-smokers only[d]	1.00 (Reference)	1.18 (0.68,2.06)	1.05 (0.40,2.77)	--[c]	0.91 (0.70,1.19)	1.00 (Reference)	00 (0.60,1.66)	1.23 (0.52,2.89)	2.70 (0.55,13.16	1.16 (0.94,1.42)

[a] Odds ratios estimated using multinomial logistic regression models with exposures and covariates multiply imputed (M=20).
[b] Models adjusted for a priori covariates of age, pre-pregnancy BMI, race/ethnicity, education, marital status, and nulliparity, plus pre-pregnancy alcohol, plasma cotinine, stress, periconception and first trimester Healthy Eating Index-2010.
[c] Women with caffeine intake >200 excluded due to lack of model convergence from a small sample size and limited cases of preeclampsia/gestational hypertension within this category.
[d] Models adjusted for a priori covariates of age, pre-pregnancy BMI, race/ethnicity, education, marital status, and nulliparity.

© 2021 Hinkle SN et al. *JAMA Network Open.*

eFigure 1. Sample Flow Chart

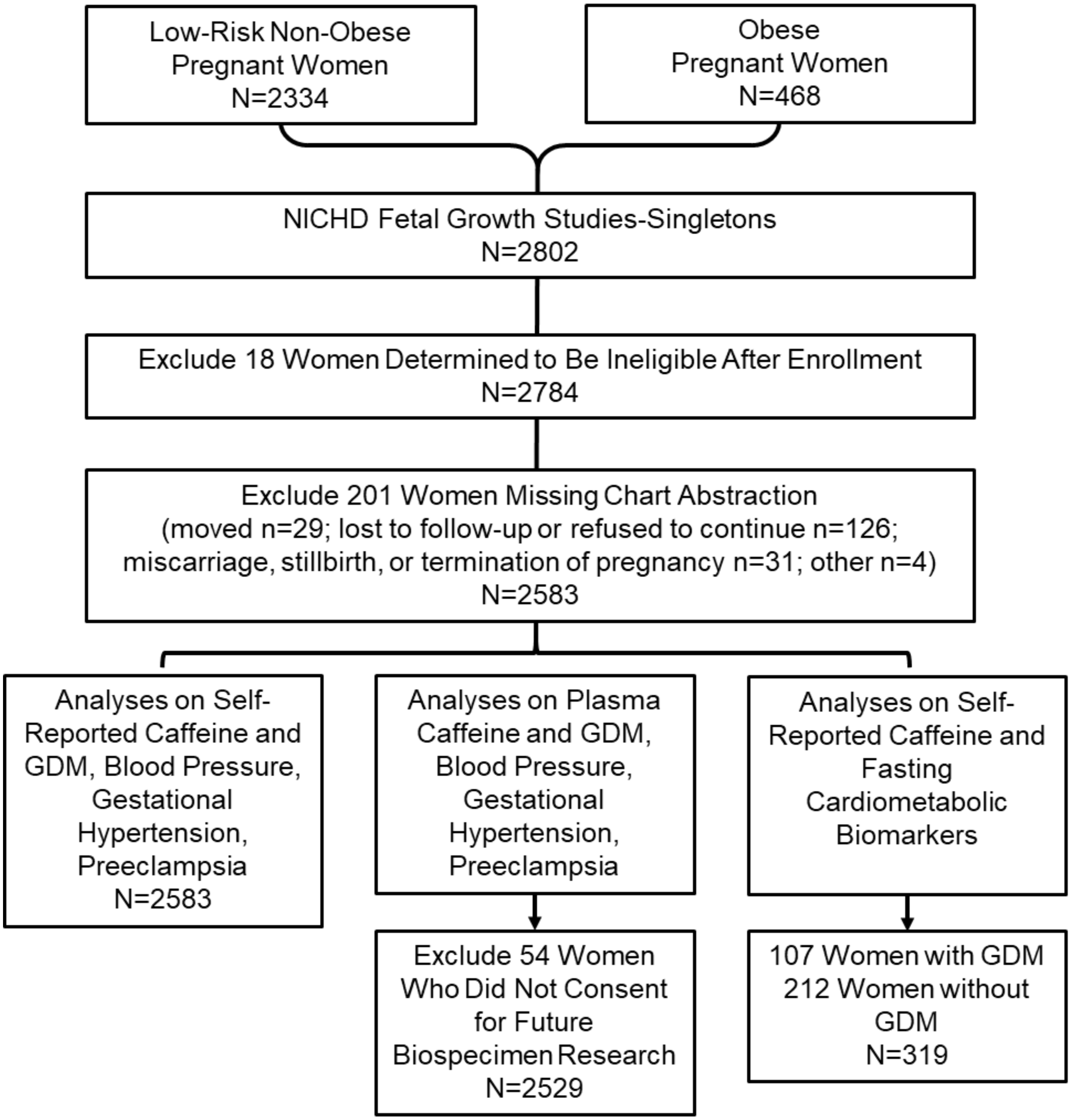

© 2021 Hinkle SN et al. *JAMA Network Open.*

eFigure 2. Prospective Adjusted Associations Between Self-reported Caffeinated Beverage Intake (mg/d) at 10 to 13 weeks and Total Plasma Caffeine and Paraxanthine at 10 to 13 Weeks Gestation and Blood Pressure Across Gestation

A. Self-reported caffeinated beverage intake (mg/d) at 10 to 13 weeks

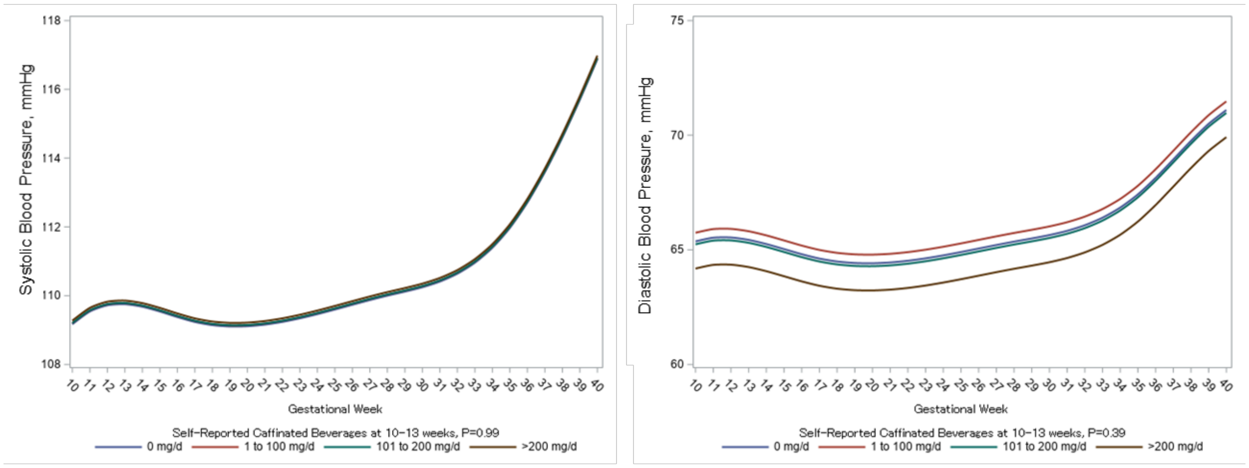

B. Total plasma caffeine and paraxanthine at 10 to 13 weeks gestation and blood pressure across gestation[a]

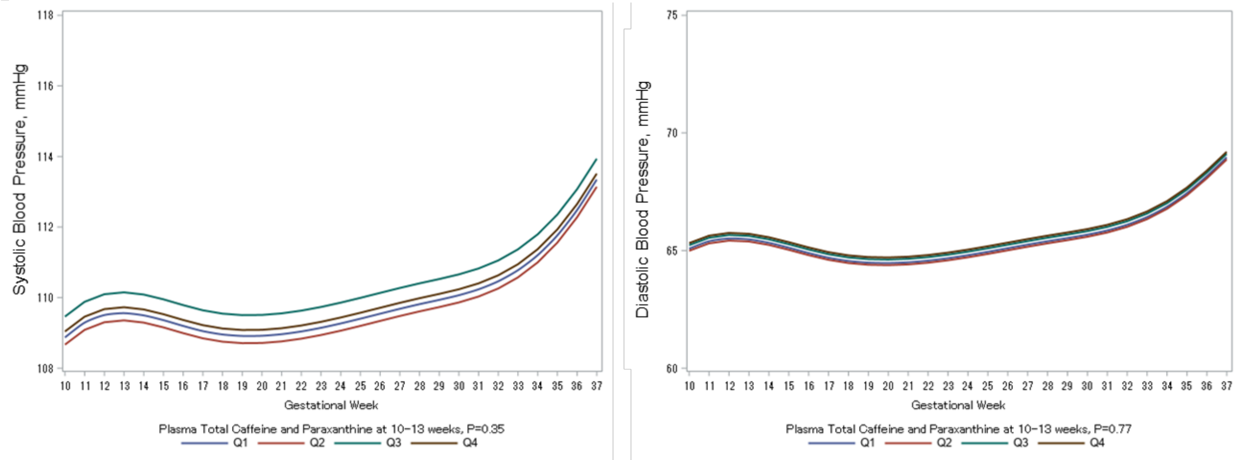

[a] Trajectories estimated from linear mixed effects models with cubic splines, adjusting for age, pre-pregnancy BMI, race/ethnicity, parity, marital status, and attained education. Estimates based on a complete case analysis: n=2512 for self-reported caffeinated beverage intake; n=2369 for plasma caffeine and paraxanthine.

© 2021 Hinkle SN et al. *JAMA Network Open.*
